# MiR-451 in Inflammatory Diseases: Molecular Mechanisms, Biomarkers, and Therapeutic Applications—A Comprehensive Review Beyond Oncology

**DOI:** 10.3390/cimb47020127

**Published:** 2025-02-16

**Authors:** Fei-Xiang Wang, Guo Mu, Zi-Hang Yu, Zhen-Shan Qin, Xing Zhao, Zu-An Shi, Xin Fan, Li Liu, Ye Chen, Jun Zhou

**Affiliations:** 1 Department of Anesthesiology, The Affiliated Hospital, Southwest Medical University, Luzhou 646000, China; 18308310180@163.com (F.-X.W.); muguo@zg120.cn (G.M.); yuzihang0114@163.com (Z.-H.Y.); 20220299120140@stu.swmu.edu.cn (Z.-S.Q.); 20220299120141@stu.swmu.edu.cn (X.Z.); szachn@163.com (Z.-A.S.); fanxin0705@swmu.edu.cn (X.F.); niuniudoctor@swmu.edu.cn (L.L.); 2 Anesthesiology and Critical Care Medicine Key Laboratory of Luzhou, Southwest Medical University, Luzhou 646000, China; 3 Department of Traditional Chinese Medicine, The Affiliated Hospital, Southwest Medical University, Luzhou 646000, China; chenye0117@swmu.edu.cn

**Keywords:** miR-451, biomarker, therapeutic target, inflammatory diseases, microRNA

## Abstract

MicroRNAs play crucial roles in regulating inflammatory responses and disease progression. Since its identification on chromosome 17q11.2 in 2005, miR-451 has emerged as a key regulator of multiple physiological and pathological processes. While its role in cancer has been extensively documented, accumulating evidence reveals miR-451’s broader significance in inflammatory conditions through the regulation of NF-κB, AMPK, and PI3K signaling pathways. This comprehensive review systematically analyzes miR-451’s multifaceted functions in inflammatory diseases, with particular focus on ischemia–reperfusion injury, arthritis, and acute organ injuries. We present compelling evidence for miR-451’s potential as a diagnostic biomarker, demonstrating its distinctive expression patterns across various biological specimens and disease states. Furthermore, we elucidate how miR-451 modulates inflammatory responses through the regulation of immune cell populations, including microglia activation, macrophage polarization, and neutrophil chemotaxis. By integrating current evidence and bioinformatic analyses, we establish a theoretical framework linking miR-451’s molecular mechanisms to its therapeutic applications. This review not only synthesizes the current understanding of miR-451 in inflammatory diseases but also provides critical insights for developing novel diagnostic tools and therapeutic strategies.

## 1. Introduction

Inflammation plays a central role in the initiation and development of a wide array of diseases. Its significant influence on the trajectory of these diseases positions it as a critical and challenging subject in medical research. This process is a protective immune response, initiated by the evolutionarily conserved innate immune system, in response to harmful stimuli including pathogens, necrotic cells, and various irritants. The response is intricately regulated by the host’s own mechanisms. An inadequate inflammatory response may result in the prolonged presence of pathogens, leading to persistent infections. Conversely, an overactive inflammatory response may precipitate chronic or systemic inflammatory disorders. Consequently, the precise regulation of the inflammatory process is vital for determining the disease outcome [1].

MicroRNAs (miRNAs), small endogenous RNAs, play a pivotal role in gene expression through post-transcriptional regulation. These molecules have attracted considerable interest in both fundamental biomedical research and translational applications due to their capacity to modulate gene expression and their ubiquitous presence across various bodily tissues and fluids. Furthermore, miRNAs are increasingly recognized for their potential as disease biomarkers. They primarily function by targeting the 3′ untranslated regions of messenger RNAs (mRNAs), thereby influencing mRNA stability and, in turn, regulating gene expression levels. Alterations in the expression of miRNAs can significantly affect their regulatory impact on target mRNAs, disrupting cellular equilibrium. Consequently, the interplay between miRNAs and mRNAs is crucial in understanding the mechanisms underlying disease onset and progression [2,3].

Since Altuvia et al. first identified the gene encoding miR-451 in the human pituitary gland in 2005, situated in the 17q11.2 region of the human chromosome, the significance of miR-451 in an array of physiological and pathological processes has been subject to increasing examination. These processes include, but are not limited to, hematopoietic differentiation, embryonic development, epithelial cell polarity, neurodevelopment, ischemia–reperfusion injury, arthritis, and multiple cancer types [4,5,6]. Despite substantial evidence delineating the intricate relationships among miR-451, inflammatory processes, and oncogenesis [7,8,9], a systematic analysis of miR-451’s regulatory roles in non-neoplastic inflammatory pathologies has not been thoroughly addressed in the literature. Therefore, this review seeks to explore the potential of miR-451 as a therapeutic target and biomarker for non-tumorous inflammatory diseases, considering a variety of inflammatory conditions based on current research, and aims to offer theoretical backing for the treatment of associated inflammatory disorders.

## 2. MiR-451 Family

The miR-144/451 cluster, located on chromosome 17q11.2, represents a unique and evolutionarily conserved genomic structure in vertebrates. This cluster consists of three key members: miR-144, miR-451a (mature sequence: 5′-AAACCGUUACCAUUACUGAGUU-3′, commonly referred to as miR-451 in the literature), and miR-451b (mature sequence: 5′-UAGCAAGAGAACCAUUACCAUU-3′). These miRNAs, while sharing genomic proximity, exhibit distinct characteristics in their sequences, biogenesis pathways, and biological functions [10,11].

What makes this cluster particularly interesting is its distinctive biogenesis pathways. While miR-144 and miR-451b follow the canonical miRNA processing pathway involving both Drosha and Dicer, miR-451a undergoes a unique Dicer-independent, Ago2-mediated maturation process. This special processing pathway is attributed to miR-451a’s unusual structure: its mature form (23 nucleotides) includes sequences from both arms of the hairpin precursor and extends through the loop region, a feature not observed in other miRNAs. The miR-451a precursor forms a short 17-nucleotide stem structure that is too short for Dicer processing but is perfectly suited for Ago2-mediated cleavage and maturation. This distinctive feature was initially discovered through observations that miR-451a effectively accumulates in Dicer-deficient cells [10,11].

The functional divergence between miR-451a and miR-451b is reflected in their expression patterns and regulatory networks. MiR-451a has been extensively documented for its roles in hematopoietic differentiation, tumor suppression, and inflammatory regulation [11,12,13]. Its expression is particularly prominent in erythroid cells and is crucial for erythropoiesis [14]. In contrast, miR-451b’s specific functions and regulatory targets remain to be fully elucidated, though its distinct sequence suggests different target specificity and potentially unique biological functions [15].

Genomically, this miRNA cluster is positioned in an intergenic region adjacent to the ERAL1 gene, which encodes a mitochondrial protein and is transcribed in the opposite direction. While ERAL1 and the miR-144/451 cluster share physical proximity in the genome, they are transcriptionally independent. Another interesting feature of this genomic region is miR-4732, which is independently regulated and follows the canonical miRNA processing pathway. The spatial organization of these genes suggests potential co-regulatory mechanisms that warrant further investigation [10,11].

**Figure 1:**
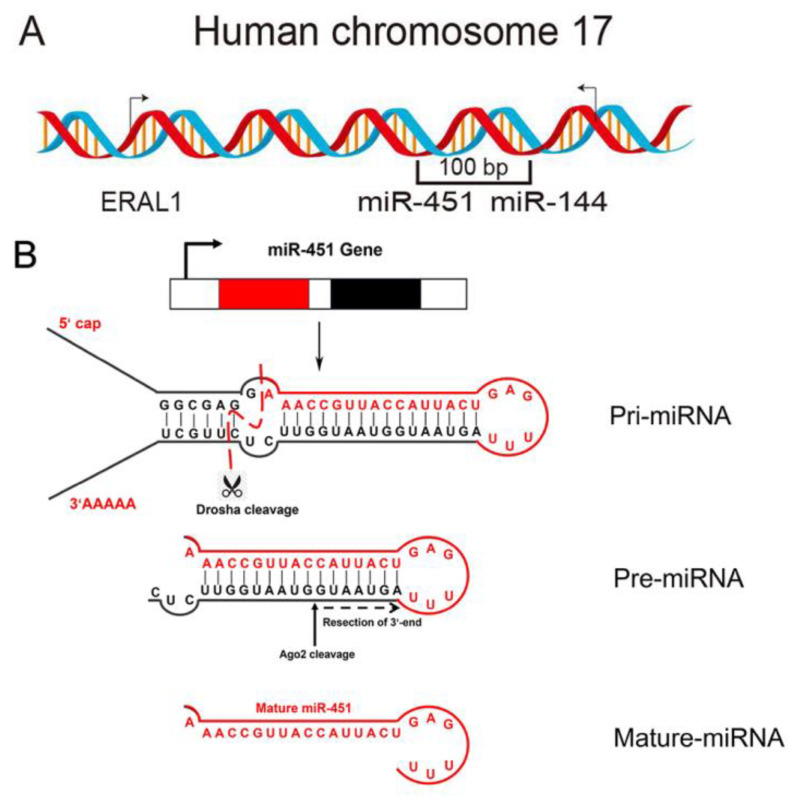
Localization and noncanonical biogenesis pathway of the miR-144/451 gene locus in the human genome. (**A**) Features of the miR-144/451 locus including the adjacent ERAL1 gene. (**B**) Model for the processing of miR-451.

The evolutionary aspects of this cluster provide additional insights into its biological significance. MiR-451a demonstrates remarkable conservation across vertebrates, suggesting fundamental biological importance [10]. In contrast, the evolutionary history and conservation patterns of miR-451b require further investigation to fully understand its phylogenetic significance and functional implications in different species [15]. The unique structural and processing characteristics of these miRNAs, particularly miR-451a’s Ago2-dependent maturation, contribute to their specific biological functions and target recognition patterns. This specificity is crucial for understanding their roles in various physiological and pathological processes, including inflammation and disease development [10,11] (Figure 1).

## 3. MiR-451 as a Diagnostic Biomarker: Current Evidence and Future Potential

**Figure 2:**
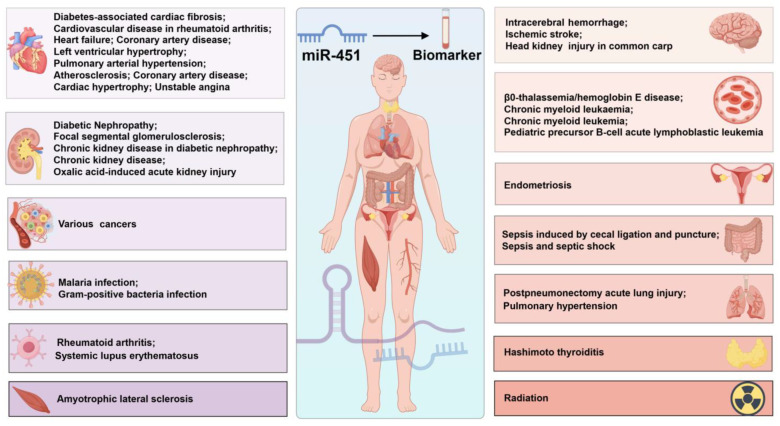
Disease spectrum associated with miR-451 as a biomarker. Studies demonstrate that miR-451 functions as a robust biomarker across a diverse array of diseases. These encompass, but are not confined to, disorders affecting the uterus, heart, kidneys, brain, intestines, lungs, multiple forms of cancer, and conditions pertaining to the hematological and immune systems.

The identification and validation of reliable biomarkers remain crucial for accurate disease diagnosis, precise therapeutic monitoring, and improved prognostic evaluation. Among various molecular markers, miR-451 has emerged as a promising candidate across multiple pathological conditions, demonstrating significant diagnostic and prognostic value (Figure 2, Table 1). Our analysis reveals that miR-451’s potential as a biomarker spans a broad spectrum of diseases, including endometriosis, diabetes-associated cardiac fibrosis, β0-thalassemia/hemoglobin E disease, diabetic nephropathy, focal segmental glomerulosclerosis, and cardiovascular complications in rheumatoid arthritis [12,13,16,17,18,19]. Moreover, a key advantage of miR-451 as a biomarker lies in its detectability across diverse biological specimens. While most current studies focus on plasma-based detection [4,10,18,19,20,21], emerging evidence demonstrates its utility in various sample types, including lesion tissues, urinary exosomes, feces, peripheral monocytes, and bone marrow aspirates [12,13,17,22,23,24]. This multi-specimen detectability enhances its potential for comprehensive disease monitoring and could facilitate more accurate diagnostic and prognostic assessments through integrated analysis of multiple sample types.

**Figure 3:**
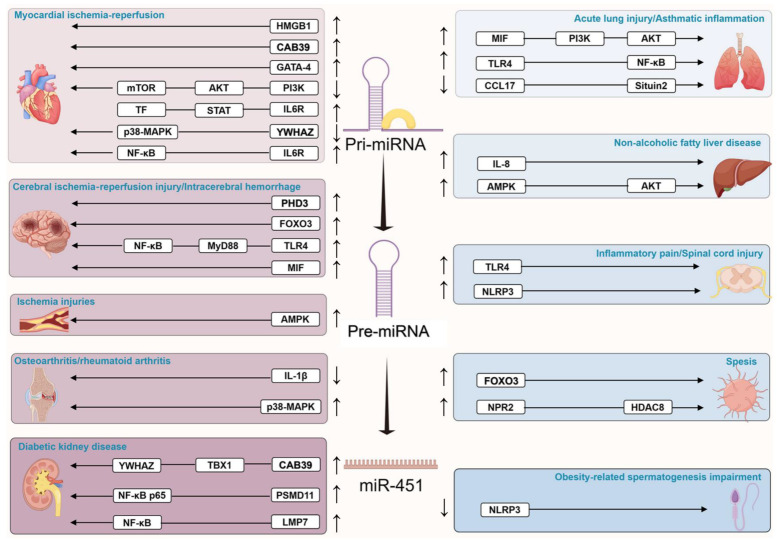
MiR-451 and its associated signaling pathways in inflammatory diseases. The figure illustrates the relationship between miR-451 and key inflammatory signaling pathways (NF-κB, AMPK, MAPK, and NLRP3) in non-tumorous inflammatory diseases. Upward arrows (↑) indicate scenarios where miR-451 exhibits protective effects with the involvement of these pathways, while downward arrows (↓) represent conditions where altered miR-451 expression is associated with disease progression through pathway-mediated mechanisms. These pathways have been implicated in mediating the biological effects of miR-451 across various inflammatory conditions, though the exact regulatory mechanisms may vary depending on the specific disease context.

The relationship between miR-451, inflammation, and disease progression is particularly noteworthy. In the context of cancer, inflammatory processes can either precede malignant transformation or emerge as a consequence of oncogenic mutations. These inflammatory conditions significantly influence disease outcomes by affecting cell proliferation, angiogenesis, metastasis, and treatment response [52]. The established links between cancer, inflammation, and miR-451 [4,10,20] extend beyond oncology, with mounting evidence suggesting its broader role in various inflammatory conditions (Figure 3, Table 2).

Overall, miR-451 has emerged as a promising diagnostic biomarker across multiple pathological conditions, with its expression patterns showing distinct characteristics based on disease types and sample origins. In systemic inflammatory disorders, including rheumatoid arthritis, Hashimoto thyroiditis, and systemic lupus erythematosus, circulating miR-451 levels are predominantly upregulated [25,26,27]. However, in tissue-specific inflammatory conditions such as endometriosis, miR-451 exhibits differential expression patterns downregulated in plasma but upregulated in lesion tissues [12], highlighting the importance of sample type selection in biomarker analysis.

The expression profile of miR-451 demonstrates disease-specific patterns, particularly in cardiovascular and renal disorders. In cardiovascular diseases, chronic conditions like heart failure and pulmonary hypertension show consistent downregulation [28,31,32], while acute conditions such as coronary artery disease and left ventricular hypertrophy typically exhibit upregulation [29,30,33,34]. In renal disorders, urinary exosomal miR-451 levels are consistently upregulated [17,38], whereas plasma levels show variable patterns [18,37], suggesting that urinary miR-451 might serve as a more reliable marker for kidney disease monitoring.

While the widespread alterations of miR-451 across various diseases might initially appear to limit its specificity as a biomarker, this characteristic can be advantageously utilized through multi-parameter diagnostic approaches. The high detectability of miR-451 across diverse biological specimens, including plasma, urinary exosomes, lesion tissues, and bone marrow aspirates [12,13,17,22,23,24], enables comprehensive disease monitoring through integrated analysis of multiple sample types. Furthermore, the temporal dynamics of miR-451 expression during disease progression and treatment response provide additional diagnostic value, particularly when combined with traditional clinical parameters [75,76,77,78].

To enhance the clinical utility of miR-451 as a biomarker, standardization of sample collection, processing methods, and data interpretation is crucial. The implementation of disease-specific cutoff values, sample-specific reference ranges, and consideration of confounding factors will be essential for reliable diagnostic applications. Additionally, the development of multi-marker panels incorporating miR-451 with other disease-specific markers may improve diagnostic accuracy and specificity, particularly in complex pathological conditions [4,10,20,52]. The subsequent sections of this review will systematically analyze the functional roles and molecular mechanisms of miR-451 in various non-tumorous inflammatory conditions, evaluating its dual potential as both a diagnostic biomarker and therapeutic target.

## 4. MiR-451 as a Therapeutic Target

### 4.1. Ischemia–Reperfusion Injury (IRI)

IRI represents a critical challenge in contemporary medical practice, significantly compromising patient safety. Extensive research has demonstrated that IRI can be precipitated by a variety of clinical conditions, including sepsis, organ transplantation, shock, myocardial infarction, cerebral ischemia, and stroke [79,80,81,82,83,84]. Interventions targeting the inflammatory processes associated with IRI have shown promise in enhancing patient outcomes. For instance, formononetin has been observed to mitigate inflammation in cerebral IRI in rodents by modulating the JAK2/STAT3 signaling pathway, illustrating the potential for targeted inflammatory therapeutic strategies in improving the prognosis of cerebral IRI [85].

Recent investigations into the connection between miR-451 and IRI have predominantly centered on the cerebral and cardiac manifestations of this condition. Evidence indicates that miR-451-3p exerts a protective influence in myocardial IRI by suppressing MAP1LC3B-mediated autophagy. This action suggests miR-451-3p as a potential novel molecular target for cardiac IRI treatment, aiming to enhance the clinical prognosis of heart disease [86]. Furthermore, miR-451 has been shown to regulate HMGB1 mRNA and protein expression at the transcriptional level. The upregulation of miR-451 leads to a reduction in HMGB1 expression, mitigating IRI and safeguarding myocardial cells, potentially through the diminution of oxidative stress injuries and the prevention of cell apoptosis [5]. In the context of cerebral IRI, microRNA-451 shields neurons from IRI-induced cell mortality by targeting CELF2. The deliberate overexpression of miR-451 diminishes ischemic cerebral infarction and apoptotic activity in mice subjected to focal ischemic stroke, while its suppression markedly intensifies ischemic harm [87,88]. Importantly, miR-451 possesses significant potential as a biomarker for IRI. For example, the peripheral blood expression levels of miR-451 have been significantly linked with the incidence of ischemic stroke, underlining its viability as a biomarker for this ailment [47]. In a similar vein, another investigation demonstrated that ischemic stroke patients exhibited substantially lower circulating miR-451 levels than control subjects. Further analysis showed an inverse correlation between miR-451 levels and both the scores on the National Institutes of Health Stroke Scale and the volume of infarction [87]. These results affirm the instrumental role of miR-451 in the IRI of vital organs such as the heart and brain, underscoring its potential as both a therapeutic target and a biomarker. Nevertheless, elucidating the impact of miR-451 on IRI in other organs necessitates additional research.

### 4.2. Arthritis

Arthritis, characterized as an inflammatory condition initiated by factors such as inflammation, infection, degeneration, trauma, or others predominantly impacts the human body’s joints and adjacent tissues. This condition encompasses several types, all of which can severely impair joint functionality and interfere with daily life [89,90,91]. Studies have underscored a profound link between inflammation and arthritis, noting that bacteria, viruses, fungi, and parasites can trigger both acute and chronic forms of the disease, including infectious/septic arthritis, reactive arthritis, and inflammatory arthritis [92].

The etiology of arthritis predominantly stems from the inflammation-driven degeneration of bone and cartilage, thereby positioning the effective management of inflammation as a pivotal element in therapeutic strategies. Investigations utilizing a bilateral anterior cruciate ligament transection model in rats have revealed elevated expression levels of miR-451, which intensify the impact of IL-1β on articular chondrocytes [66]. In addition, miR-451 has been demonstrated to alleviate the severity of rheumatoid arthritis and diminish the prevalence of infiltrating cells by curtailing neutrophil chemotaxis via the p38 MAPK signaling pathway [93]. Conversely, evidence suggests that the application of miR-451 inhibitors may ameliorate osteoarthritis severity in rats, indicating a potential differential role of miR-451 across various arthritis forms [6]. This necessitates additional studies to elucidate its precise mechanisms. Moreover, miR-451 is implicated in the pathophysiology of other arthritis variants. Elevated expressions of miR-21, miR-451, miR-223, and miR-16 have been identified not solely in rheumatoid arthritis patients but also in those diagnosed with systemic lupus erythematosus [27]. Significantly, in subjects predisposed to rheumatoid arthritis, an upsurge in miR-451 expression within peripheral blood mononuclear cells underscores its critical role in the disease’s prophylaxis [94]. Given its regulatory function over CXCL16, miR-451 is posited to influence the course of arthritis by modulating immune cell recruitment [94]. Nonetheless, comprehensive investigations are imperative to dissect the intricate roles of miR-451 in arthritis and assess its viability as a therapeutic target, thereby elucidating its definitive function in arthritis management.

### 4.3. Acute Lung Injury

The respiratory system is pivotal in upholding the body’s normal operational functions, with the lungs frequently encountering damage as the initial site of injury in various conditions. Acute lung injury (ALI), a concept with over five decades of clinical history, has been identified as a major influence on the morbidity and mortality rates among critically ill patients. Neutrophils are instrumental in this context, serving as a universal conduit to the loss of vascular integrity, irrespective of their direct association with the injury. Consequently, interventions focusing on the neutrophil-driven inflammatory response and preservation of vascular integrity are vital strategies in the management of ALI [95].

MiR-451 demonstrates significant therapeutic promise for ALI management. Research has shown that the depletion of the miR-144/451 cluster intensifies sepsis-induced oxidative harm in lung epithelial cells [72]. Moreover, exosomes from human umbilical cord mesenchymal stem cells, enriched with miR-451, facilitate macrophage polarization towards the M2 phenotype via the modulation of the MIF-PI3K-AKT signaling cascade, thus offering relief from ALI symptoms [68]. In addition, miR-451-loaded exosomes adeptly modulate the secretion levels of pro-inflammatory cytokines, including TNF-α, IL-1β, and IL-6, through the TLR4/NF-κB signaling pathway, contributing to the reduction in ALI’s inflammatory responses [69]. Further investigations have unveiled that elevated miRNA-451 expression in rat pulmonary endothelial cells augments cell permeability while concurrently suppressing angiogenesis, highlighting its potential in addressing burn-related injuries [96]. MiR-451 also plays a crucial role in bronchopulmonary dysplasia regulation by affecting macrophage migration inhibitory factors [97]. Within the sphere of chronic lung inflammation, it adeptly adjusts macrophage phenotypes triggered by allergens, thus moderating allergic asthma’s inflammatory processes [70]. Considering inflammation, immune cell infiltration, and compromised pulmonary vascular integrity as pivotal factors in ALI’s etiology, and given miR-451’s regulatory capacity over these mechanisms, targeting miR-451 emerges as a viable and promising approach in the therapeutic landscape of ALI.

### 4.4. Acute Kidney Injury (AKI)

Acute KI (AKI) encompasses a diverse array of disorders precipitated by various causative factors, all of which are marked by an abrupt decline in glomerular filtration rate. This decline is manifest through elevated serum creatinine levels or diminished urine output [98]. The role of inflammation is critical in the initiation and progression of AKI, particularly when it is associated with sepsis. Moreover, although the extant diagnostic criteria for AKI constrain the possibilities for early identification, recent research has underscored the utility of innovative biomarkers related to renal stress and injury. These biomarkers hold promise for assessing the risk of sepsis-related AKI and enabling early diagnosis. Therefore, addressing inflammation and uncovering relevant biomarkers are crucial for the effective diagnosis and management of AKI [99].

MiR-451 exhibits a beneficial biological role in the context of AKI. Studies have demonstrated that damage to renal cells triggers the release of exosomes containing miR-451, which is associated with a decline in human renal function [38]. Moreover, miR-451 is implicated in the mediation of cadmium-induced damage to the cephalic kidneys of common carp, specifically targeting cacna1ab via autophagy pathways [49]. In Tallyho/Jng mice, the systemic suppression of miR-451 leads to enhanced fibrotic signaling and reduced autophagic activity, thus aggravating renal damage [100]. Additionally, miR-451 acts as a circulating microRNA biomarker for oxalate-induced AKI [39]. Beyond its immediate impact, miR-451 is also recognized for its potential involvement in CKD, including diabetic nephropathy and chronic kidney disease in pediatric patients with β-thalassemia [63,101]. Through the regulation of inflammation-associated pathways, such as NF-κB, miR-451 contributes to the advancement of these conditions and is considered a promising candidate for therapeutic targeting and biomarker development [63].

### 4.5. Nonalcoholic Steatohepatitis (NASH)

NASH represents an inflammatory variant of non-alcoholic fatty liver disease (NAFLD) linked to disease advancement, cirrhosis development, and a heightened necessity for liver transplantation [102]. A critical challenge in this domain involves identifying the inflammatory triggers that facilitate the progression from NAFLD to NASH. The sources of hepatic inflammation can be both extrinsic and intrinsic to the liver, encompassing factors like lipotoxicity, innate immune responses, cell death pathways, mitochondrial dysfunction, and endoplasmic reticulum stress, all contributing to NASH development [103]. Therefore, implementing anti-inflammatory approaches and fostering the resolution of inflammation stand as principal therapeutic objectives in NASH management.

It is significant that microRNA-451 demonstrates substantial therapeutic potential in managing NASH. Research has shown that microRNA-451 downregulation in NASH can inhibit the generation of fatty acid-induced pro-inflammatory cytokines through the AMPK/AKT signaling pathway [65]. Moreover, trans-Chalcone not only mitigates insulin resistance and hepatic inflammation but also facilitates the elimination of cholesterol in the liver of high-fat diet-induced rats. These effects are intricately linked to its modulatory actions on the miR-34a, miR-451, and miR-33a pathways [64]. Additionally, studies have underscored that the combined application of MicroRNA-451 and Genistein ameliorates NASH symptoms in mice [104]. We strongly believe that ongoing research will further elucidate the complex relationship between miR451 and NASH in more comprehensive detail.

### 4.6. Inflammatory Pain

Acute inflammation functions as an inherent physiological response to tissue injury or infection, contrasting with chronic inflammation, which is maladaptive and often culminates in pronounced discomfort. Chemical mediators responsible for triggering tissue inflammation interact with nociceptive nerve endings. This interaction decreases the excitation threshold of neurons and increases the rate of afferent nerve firing, which subsequently gives rise to allodynia and hyperalgesia [105]. Inflammatory conditions and neuropathic damage activate signaling pathways that frequently precipitate severe and persistent pain syndromes in affected individuals [106]. The intricate relationship between pain and inflammation underscores that inflammatory pain profoundly affects patients’ quality of life. Hence, addressing both pain and the underlying inflammation is vital for the effective management and prognosis of inflammatory pain conditions.

Research has shown that elevated levels of miR-451 can mitigate inflammatory pain by inhibiting the inflammatory responses triggered by microglial activation. This effect is linked to miR-451’s capability to target TLR4 specifically [71]. Moreover, it is important to highlight the considerable promise of miR-451 as a biomarker for unstable angina [36]. Further investigations have also revealed that miR-451 plays a role in the progression of coronary artery disease via the PI3K-Akt-mTOR signaling pathway and its interaction with VEGFA [33]. Considering VEGFA’s pivotal function in a myriad of vascular diseases, this emphasizes the significant role of miR-451 within vascular disease mechanisms. Nevertheless, to firmly establish these insights, more extensive research is required.

### 4.7. Sepsis

Sepsis is triggered by an aberrant host response to infection, precipitating life-threatening organ dysfunction. This condition has emerged as a critical contributor to patient mortality in intensive care units and escalates healthcare expenses [107]. Despite substantial progress in sepsis treatment in recent years, its incidence and mortality rates persistently rise. The heterogeneous clinical presentations of sepsis pose significant diagnostic, therapeutic, and management challenges for healthcare professionals [108]. Consequently, curtailing the development of sepsis-associated systemic inflammatory response syndrome and multiple organ dysfunction syndrome, along with the identification of more accurate biomarkers, is essential for effective sepsis diagnosis and therapy [109].

It is noteworthy that miR-451 appears to play a pivotal role in the onset and progression of sepsis. Data from experiments indicate that the deletion of the miR-144/451 cluster intensifies oxidative stress and apoptosis in lung epithelial cells triggered by sepsis [72]. Furthermore, analysis employing cecal ligation and puncture techniques to investigate the expression of circulating microRNAs in experimental sepsis revealed a pronounced increase in miR-451 levels [42]. Additionally, research has shown that in murine whole blood exposed to lipopolysaccharide (LPS), miR-451 is significantly upregulated. This enhancement in expression correlates with both the dose and duration of LPS exposure [43]. We posit that ongoing research will further delineate the intricate and comprehensive relationship between miR-451 and sepsis.

### 4.8. Other Inflammation-Related Diseases

Research has demonstrated that miR-451 is implicated in a diverse array of inflammatory conditions beyond the primary diseases under discussion. These include endothelial cell inflammation, inflammatory bowel disease, microglial inflammation, NLRP3 inflammasome-related spermatogenesis, inflammation related to grass carp, Hashimoto thyroiditis, and acute graft-versus-host disease, among others [55,73,74,110,111,112,113]. The substantial link between miR-451 and a wide range of inflammation-related diseases, coupled with findings that modulating miR-451 can effectively slow disease progression and facilitate therapeutic benefits, underscores the vast potential of miR-451 as both a biomarker and a therapeutic target for inflammation-related conditions. This assertion, however, warrants further investigation for more definitive evidence.

## 5. Target Gene Prediction and Enrichment Analysis for miR-451

**Figure 4:**
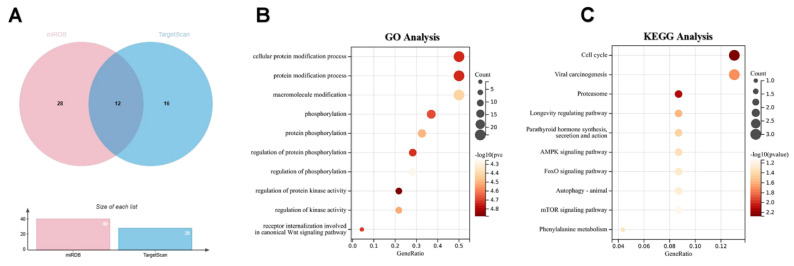
Predicted target genes and pathway enrichment analysis of hsa-miR-451a. (**A**) Target genes of hsa-miR-451a predicted using the miRDB and TargetScan databases. (**B**) Gene ontology (GO) analysis of the predicted target genes associated with hsa-miR-451a. (**C**) Kyoto Encyclopedia of Genes and Genomes (KEGG) pathway analysis of the predicted target genes for hsa-miR-451a.

The aforementioned results confirm that miR-451 is closely related to inflammatory diseases, predominantly through the action of miR-451a. To further explore its mechanisms of action, we utilized TargetScan (www.targetscan.org) and miRDB (mirdb.org) to predict potential target genes for hsa-miR-451a. A comparative analysis across these platforms, supported by Venn diagrams [114], identified critical genes such as OSR1, PSMB8, CDKN2D, MIF, SAMD4B, CAB39, VAPA, PMM2, S1PR2, FBXO33, ATF2, and TTN. Further gene ontology (GO) analysis indicated that hsa-miR-451 predominantly influences cellular protein modification processes, with a particular focus on phosphorylation. Enrichment analysis using Kyoto Encyclopedia of Genes and Genomes (KEGG) pathways revealed significant links between hsa-miR-451 and several biological pathways including the cell cycle, viral carcinogenesis, proteasome, AMPK, and mTOR pathways. These findings align with existing research [58,65,76], underscoring the critical role of miR-451 in inflammatory diseases and its potential as a therapeutic target and biomarker (Figure 4).

## 6. Conclusions and Perspectives

The extensive research reviewed here demonstrates that miR-451 plays multifaceted roles in inflammatory diseases through several key mechanisms. First, miR-451 functions as a master regulator of inflammatory pathways, particularly through modulation of NF-κB, AMPK, and PI3K signaling cascades, which are crucial in inflammatory disease progression [62,63,65,68,76]. Our systematic analysis reveals that miR-451 predominantly exerts its effects by targeting key molecules within these pathways, including HMGB1 in myocardial IRI [5], MIF in ALI [68], and TLR4 in inflammatory pain [71], thereby influencing cellular responses to inflammatory stimuli.

Second, miR-451 demonstrates remarkable versatility in regulating various immune cell populations. Current evidence shows its capacity to modulate microglia activation [55], macrophage polarization [68], and neutrophil chemotaxis [93], suggesting a broader role in orchestrating immune responses. This immune-regulatory function is particularly evident in conditions such as ALI, where miR-451-containing exosomes from mesenchymal stem cells facilitate M2 macrophage polarization through the MIF-PI3K-AKT signaling pathway [68], and in rheumatoid arthritis, where it modulates neutrophil recruitment through p38 MAPK signaling [93]. Furthermore, miR-451 has shown promising effects in alleviating inflammatory responses in multiple organs, including the lung [68], brain [87,88], and heart [86].

Third, the potential of miR-451 as a biomarker has been extensively validated across multiple inflammatory conditions. Our comprehensive review of the literature reveals distinctive expression patterns of miR-451 in various biological fluids and tissues, with notable consistency in certain pathological states. For instance, miR-451 shows consistent downregulation in cardiovascular conditions [19,28,31,32] and upregulation in several autoimmune disorders [25,26,27]. The detection of miR-451 in various biological samples, including plasma [12,19], tissue [12,13], urinary exosomes [17,38], and peripheral blood mononuclear cells [94], demonstrates its versatility as a diagnostic tool.

Several promising research directions emerge from current findings. Exosome-based therapeutic applications: Given the success of miR-451-containing exosomes in treating ALI [68,69], future research should explore this delivery method for other inflammatory conditions. Of particular interest is the potential application in disorders where traditional drug delivery faces barriers, such as cerebral inflammation [87,88] or autoimmune disorders [93,94]. The unique properties of exosomes in crossing biological barriers while protecting their miRNA cargo make this an especially promising avenue for therapeutic development.

Tissue-specific targeting mechanisms: While we understand many of miR-451’s molecular targets, such as HMGB1 [5], NF-κB [53], and TLR4 [71], the tissue-specific mechanisms determining its efficiency and specificity remain unclear. Future studies should investigate how tissue context influences miR-451’s function and whether tissue-specific delivery systems could enhance therapeutic efficacy. This is particularly relevant given the diverse effects observed in different organs, from neuroprotection [87,88] to cardioprotection [86].

Cross-talk between organs: An intriguing question emerges regarding miR-451’s potential role as an inter-organ messenger, particularly given its presence in circulation [42,43] and exosomes [68,69]. Investigation is needed to determine whether miR-451 released from damaged organs can affect other organs, particularly in conditions like sepsis where multiple organ dysfunction occurs [72]. This could provide insights into disease progression and potential therapeutic interventions. The role of miR-451 in the miR-144/451 cluster [72] and its interactions with other regulatory molecules should be further explored in this context.

Regulatory networks: Further research should focus on understanding the complex interplay between miR-451 and other miRNAs. For example, the discovered interaction between miR-451 and miR-122 in articular chondrocytes [66] suggests the existence of broader regulatory networks. Similarly, the coordination between miR-451 and other inflammatory mediators in conditions such as NASH [104] and arthritis [93,94] warrants deeper investigation.

Biomarker optimization: While miR-451’s potential as a biomarker is clear [47,87,94], standardization of detection methods and establishment of clinically relevant thresholds are needed. This is particularly important given the variable expression patterns observed in different diseases and sample types (Table 1). Future studies should focus on large-scale validation and the development of practical diagnostic tools.

From a therapeutic perspective, several key considerations emerge: The development of targeted delivery systems for miR-451 mimics or inhibitors, considering the tissue-specific requirements of different inflammatory conditions. This is particularly relevant given the success observed in specific organ systems such as the lungs [68,69], brain [87,88], and joints [93,94].

Potential off-target effects have been investigated, given miR-451’s involvement in multiple physiological processes and its regulation of various signaling pathways including NF-κB [63], AMPK [65], and MAPK [93]. The comprehensive understanding of these pathways (Figure 3) suggests the need for careful monitoring of systemic effects.

Dosing regimens and delivery timing, particularly in acute inflammatory conditions where intervention windows may be critical, have been optimized. This is especially relevant in conditions such as IRI [86,87] and sepsis [72], where the timing of the intervention can significantly impact outcomes.

Combination therapies incorporating miR-451 modulators with existing anti-inflammatory treatments, considering the synergistic effects observed with compounds such as genistein in NASH [104] and formononetin in cerebral IRI [85] have been developed.

The evolutionary conservation of miR-451 across vertebrates, along with its complex regulatory network revealed through bioinformatics analysis (Figure 4), suggests fundamental biological importance that may extend beyond currently known functions. Target gene prediction and pathway enrichment analysis have identified critical genes such as OSR1, PSMB8, CDKN2D, and MIF along with key pathways including cell cycle regulation, AMPK signaling, and mTOR signaling. These findings provide a theoretical framework for future research directions.

Intriguingly, a critical examination of the current literature reveals several notable inconsistencies in miR-451 expression patterns across similar pathological conditions. In cardiovascular diseases, while studies have reported downregulation of miR-451 in heart failure and pulmonary hypertension [28,31,32], others have demonstrated its upregulation in coronary artery disease and left ventricular hypertrophy [29,30,33,34]. Similar discrepancies are observed in inflammatory conditions, where miR-451 shows upregulation in systemic inflammatory disorders like rheumatoid arthritis and systemic lupus erythematosus [25,26,27] but exhibits variable expression in tissue-specific inflammation [12]. These apparent contradictions might be attributed to several key factors. The temporal aspect of sample collection relative to disease progression appears crucial, particularly in distinguishing between acute and chronic conditions. The choice of biological sample type significantly influences expression patterns, as evidenced in endometriosis where miR-451 shows contrasting expression between plasma and lesion tissues [12]. Furthermore, the underlying disease mechanisms and cellular contexts may differently affect miR-451 regulation, exemplified by its varying expression in chronic kidney disease between urinary exosomes and plasma samples [37,38]. Understanding these context-dependent variations is crucial for both accurate biomarker development and therapeutic applications. These findings emphasize the need for more standardized protocols that consider disease stage, sample type, and analytical methods in future studies. Such standardization would not only help resolve current discrepancies but also enhance the reliability of miR-451 as a diagnostic tool and therapeutic target.

Moreover, despite the promising potential of miR-451 in both diagnostic and therapeutic applications, several critical challenges need to be addressed for successful clinical translation. A primary concern is the stability and standardization of miR-451 detection in biological fluids. While studies have demonstrated its detectability across various sample types, including plasma, tissue, and urinary exosomes [12,13,17,22,23,24], significant variations in expression patterns between different sample types highlight the need for standardized protocols. For instance, in endometriosis, the contrasting expression patterns observed in plasma versus lesion tissue [12] underscore the complexity of using miR-451 as a biomarker and the importance of sample type selection in clinical applications.

In the context of inflammatory regulation, miR-451 demonstrates unique characteristics compared to other well-studied inflammatory miRNAs. While miR-146a-5p primarily functions through TRAF6 signaling [115], and miR-155 specifically targets immune cell function in sepsis [116], miR-451 exhibits a broader regulatory profile. This extensive involvement across multiple inflammatory pathways, including NF-κB, AMPK, and PI3K signaling [62,63,65,68,76], suggests both advantages and challenges for therapeutic development. The broader spectrum of action might offer more comprehensive therapeutic effects but also raises concerns about potential off-target effects that require careful evaluation.

The development of effective delivery systems represents another crucial challenge in translating miR-451-based therapies. While exosome-based delivery systems have shown promise in experimental settings [68,69], issues regarding scalability, standardization, and tissue-specific targeting remain to be resolved. The challenge is particularly significant in conditions affecting multiple organs or requiring sustained therapeutic effects [72]. Additionally, the potential immunogenicity of delivery vehicles and the necessity for repeated administration need thorough investigation.

To enhance the clinical utility of miR-451, several standardization aspects require attention. These include developing validated protocols for miR-451 quantification across different biological samples, establishing reference ranges that account for demographic and clinical variables, and implementing robust quality control measures for miR-451-based therapeutic products [17,38,68,69]. Future research should focus on developing novel delivery systems that can enhance stability and tissue-specific targeting, establishing comprehensive safety profiles for long-term miR-451 modulation, and conducting large-scale validation studies to standardize detection protocols.

The resolution of these challenges will be crucial for realizing the full potential of miR-451 in clinical applications. Given the complex nature of inflammatory diseases and the diverse regulatory roles of miR-451, a multifaceted approach combining improved delivery systems, standardized detection methods, and careful consideration of individual patient factors will be essential for successful therapeutic implementation.

In conclusion, while significant progress has been made in understanding miR-451’s role in inflammatory diseases, numerous questions remain. Addressing these questions through rigorous research will be crucial for developing effective miR-451-based therapies and diagnostic tools. The field’s future lies in unraveling the complex regulatory networks involving miR-451 and translating this knowledge into practical clinical applications. The potential for miR-451 to serve as both a therapeutic target and diagnostic biomarker across various inflammatory conditions makes it an exciting focus for continued research and development.

## Figures and Tables

**Table 1:** Expression profile of miR-451 as a potential biomarker across various pathological conditions.

Disease Category	Specific Condition	Species	Biological Sample	Expression Pattern	References
Inflammatory disorders					
	Endometriosis	Human	Plasma/Lesion tissue	Downregulated/ Upregulated	[12]
	Rheumatoid arthritis	Human	Plasma	Upregulated	[25]
	Hashimoto thyroiditis	Human	Serum	Upregulated	[26]
	Systemic lupus erythematosus	Human	Plasma	Upregulated	[27]
Cardiovascular diseases					
	Cardiovascular disease in rheumatoid arthritis patients	Human	Plasma	Downregulated	[19]
	Heart failure	Human	Plasma	Downregulated	[28]
	Coronary artery disease	Human	Plasma	Upregulated	[29]
	Left ventricular hypertrophy	Human	Plasma	Upregulated	[30]
	Congenital heart disease-associated pulmonary arterial hypertension	Human	Serum	Downregulated	[31]
	Pulmonary hypertension	Human/Mouse	Plasma	Downregulated	[32]
	Coronary artery disease	Human	Blood/Plasma	Upregulated	[33,34]
	Cardiac hypertrophy	Rat	Lesion tissue	Downregulated	[35]
	Unstable angina	Human	Serum	Downregulated	[36]
Renal disorders					
	Diabetic nephropathy	Rat	Urinary exosomes	Upregulated	[17]
	Focal segmental glomerulosclerosis	Human	Plasma	Downregulated	[18]
	Chronic kidney disease in diabetic nephropathy	Human	Urine/Plasma	Downregulated/ Upregulated	[37]
	Chronic kidney disease	Human/Rat	Urinary exosomes	Upregulated	[38]
	Oxalic acid-induced acute kidney injury	Human	Serum	Downregulated	[39]
Hematological disorders					
	β0-thalassemia/hemoglobin E disease	Human	Plasma	Upregulated	[16]
	Chronic myeloid leukemia	Human	Blood/Plasma	Downregulated/ Upregulated	[40,41]
	Pediatric precursor B-cell acute lymphoblastic leukemia	Human	Bone marrow	Downregulated	[24]
Infectious diseases					
	Malaria infection	Human	Plasma	Downregulated	[21]
	Sepsis	Mouse	Blood	Upregulated	[42]
	Sepsis and septic shock	Mouse	Blood	Upregulated	[43]
	Gram-positive bacterial infection	Mouse	Blood	Upregulated	[44]
Neurological disorders					
	Intracerebral hemorrhage	Mouse	Lesion tissue	Downregulated	[45]
	Ischemic stroke	Human/Mouse	Lesion tissue/Blood	Upregulated	[46,47]
	Amyotrophic lateral sclerosis	Human	Leukocytes/Monocytes	Downregulated/Upregulated	[23]
Other conditions					
	Diabetes-associated cardiac fibrosis	Human	Lesion tissue	Upregulated	[13]
	Various cancers	Human/Dog	Tumor tissues/Plasma/Feces	Downregulated/ Upregulated	[20,22]
	Postpneumonectomy acute lung injury	Porcine	Lesion tissue	Upregulated	[48]
	Cadmium-induced head kidney injury	Carp	Lesion tissue	Downregulated	[49]
	Atherosclerosis	Human	Serum	Downregulated	[50]
	Radiation exposure	Human	Serum	Downregulated	[51]

**Table 2:** MiR-451 as a therapeutic target in non-tumorous inflammatory diseases.

Disease Category	Specific Condition	Model System	Target Pathway/Molecule	References
Central Nervous System Disorders				
	Intracerebral hemorrhage	Mouse	FOXO3	[45]
	Ischemic stroke	Mouse	PHD3	[46]
	Cerebral ischemia–reperfusion injury	Human/Rat	MIF/TLR4-MyD88-NFκB	[53,54]
	Spinal cord injury	Microglial cells	NLRP3	[55]
Cardiovascular Diseases				
	Coronary artery disease	HUVECs	PI3K-AKT-mTOR	[33]
	Myocardial ischemia–reperfusion injury	Rat/Mouse	CAB39/HMGB1/GATA-4	[5,56,57]
	Ischemic injuries	HUVECs	AMPK	[58]
	Vascular endothelial dysfunction	Rat	IL6R-STAT-TF	[59]
	Vascular injury	Rat	YWHAZ-p38 MAPK	[60]
	Atherosclerotic lesions	Rat	IL6R-NFκB	[61]
Metabolic Disorders				
	Diabetic kidney disease	Rat	CAB39-TBX1-YWHAZ	[38]
	Diabetic nephropathy	Mouse/GMCs	LMP7-NFκB/PSMD11-NFκB p65	[62,63]
	Non-alcoholic fatty liver disease	Rat/Mouse	IL-8/AMPK-AKT	[64,65]
Inflammatory Conditions				
	Osteoarthritis	Rat articular chondrocytes	IL-1β	[66]
	Rheumatoid arthritis	Human	p38 MAPK	[67]
	Acute lung injury	Rat	MIF-PI3K-AKT	[68]
	Burn-induced acute lung injury	Rat	TLR4-NFκB	[69]
	Asthmatic inflammation	Mouse	CCL17-Sirtuin2	[70]
	Inflammatory pain	Mouse	TLR4	[71]
	Sepsis	Mouse	FOXO3	[72]
	Septicemia	Grass carp	NPR2-HDAC8	[73]
Reproductive Disorders				
	Obesity-related spermatogenesis impairment	Human/Mouse	NLRP3	[74]

**Abbreviations:** AMPK, AMP-activated protein kinase; AKT, protein kinase B; CAB39, calcium binding protein 39; CCL17, C-C motif chemokine ligand 17; FOXO3, forkhead Box O3; GATA-4, GATA binding protein 4; GMCs, glomerular mesangial cells; HDAC8, histone deacetylase 8; HMGB1, high mobility group box 1; HUVECs, human umbilical vein endothelial cells; IL-1β, interleukin 1 beta; IL6R, interleukin 6 receptor; IL-8, interleukin 8; LMP7, low molecular mass polypeptide 7; MAPK, mitogen-activated protein kinase; MIF, macrophage migration inhibitory factor; mTOR, mechanistic target of rapamycin; MyD88, myeloid differentiation primary response 88; NFκB, nuclear factor kappa-light-chain-enhancer of activated B cells; NLRP3, NLR family pyrin domain containing 3; NPR2, natriuretic peptide receptor 2; PHD3, prolyl hydroxylase domain-containing protein 3; PI3K, phosphoinositide 3-kinase; PSMD11, proteasome 26S subunit, non-ATPase 11; STAT, signal transducer and activator of transcription; TBX1, T-Box transcription factor 1; TF, transcription factor; TLR4, toll-like receptor 4; YWHAZ, 14-3-3ζ.

## Abbreviations

The following abbreviations are used in this manuscript:

AKT	Protein kinase B
ALI	Acute Lung Injury
AMPK	AMP-activated protein kinase
CAB39	Calcium binding protein 39
CCL17	C-C motif chemokine ligand 17
FOXO3	Forkhead Box O3
GATA-4	GATA binding protein 4
GO	Gene ontology
HDAC8	Histone deacetylase 8
HMGB1	High mobility group box 1
IL-1β	Interleukin 1 beta
IL6R	Interleukin 6 receptor
IL-8	Interleukin 8
IRI	Ischemia–reperfusion injury
KEGG	Kyoto encyclopedia of genes and genomes
AKI	Acute kidney injury
LMP7	Low molecular mass polypeptide 7
LPS	Lipopolysaccharide
MAPK	Mitogen-activated protein kinase
MIF	Macrophage migration inhibitory factor
MiRNAs	MicroRNAs
mRNAs	Messenger RNAs
mTOR	Mechanistic target of rapamycin
MyD88	Myeloid differentiation primary response 88
NAFLD	Non-alcoholic fatty liver disease
NASH	Nonalcoholic steatohepatitis
NFκB	Nuclear factor kappa-light-chain-enhancer of activated B cells
NLRP3	NLR family pyrin domain containing 3
NPR2	Natriuretic peptide receptor 2
PHD3	Prolyl hydroxylase domain-containing protein 3
PI3K	Phosphoinositide 3-Kinase
PSMD11	Proteasome 26S subunit, non-ATPase 11
STAT	Signal transducer and activator of transcription
TBX1	T-Box transcription factor 1
TF	Transcription factor
TLR4	Toll-like receptor 4
YWHAZ	14-3-3ζ
